# The aminoindanol core as a key scaffold in bifunctional organocatalysts

**DOI:** 10.3762/bjoc.12.50

**Published:** 2016-03-14

**Authors:** Isaac G. Sonsona, Eugenia Marqués-López, Raquel P Herrera

**Affiliations:** 1 Laboratorio de Organocatálisis Asimétrica, Departamento de Química Orgánica, Instituto de Síntesis Química y Catálisis Homogénea (ISQCH) CSIC-Universidad de Zaragoza, C/ Pedro Cerbuna 12, 50009 Zaragoza, Spain

**Keywords:** aminocatalysis, 1,2-aminoindanol, bifunctional, organocatalysis, hydrogen bonding

## Abstract

The 1,2-aminoindanol scaffold has been found to be very efficient, enhancing the enantioselectivity when present in organocatalysts. This may be explained by its ability to induce a bifunctional activation of the substrates involved in the reaction. Thus, it is easy to find hydrogen-bonding organocatalysts ((thio)ureas, squaramides, quinolinium thioamide, etc.) in the literature containing this favored structural core. They have been successfully employed in reactions such as Friedel–Crafts alkylation, Michael addition, Diels–Alder and aza-Henry reactions. However, the 1,2-aminoindanol core incorporated into proline derivatives has been scarcely explored. Herein, the most representative and illustrative examples are compiled and this review will be mainly focused on the cases where the aminoindanol moiety confers bifunctionality to the organocatalysts.

## Introduction

**Figure 1:**
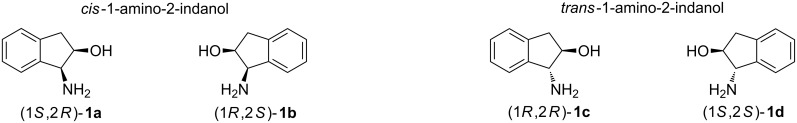
Different configurations of 1,2-aminoindanol **1a**–**d**.

The structural and chemical properties of the 1,2-aminoindanol scaffold **1** have transformed aminoindanol derivatives into versatile building blocks for the construction of catalysts and the efficient induction of chirality in asymmetric processes (Figure 1). Some examples of these properties are rigidity, disposition of the two stereogenic centers, ability of the hydroxy and amino groups to coordinate to some metals or to act as hydrogen-bond donors/acceptors, the different catalytic activity of these chemical groups and their possible derivatization. Thus, in the last decade, it has been widely employed in the field of asymmetric catalysis. Regarding the use of aminoindanol derivatives as ligands in organometallic catalytic complexes, the results have been outstanding. Examples are found in (a) the vanadium-catalyzed asymmetric oxidation of disulfides and sulfides, which are involved in the synthesis of ligands and pharmaceutical chiral synthetic precursors [1–2] and in (b) the transfer-hydrogenation reaction catalyzed by bifunctional chiral ruthenium complexes, employed in the synthesis of peptide mimics with an interesting trifluoroethylamine moiety [3–5]. However, it is in the field of asymmetric organocatalysis [6–8] where the aminoindanol core has gained more importance, being a recurrent structural motif in several organocatalytic species. Some examples are (a) the enantioselective reduction of ketones through the in situ formation of catalytically active oxazaborolidines using *cis*-1,2-aminoindanol derivatives [9–10] and (b) the synthesis of more active cooperative thiourea-urea-based organocatalysts, which employ the aminoindanol framework as structural linker between two hydrogen-bond-donor moieties [11]. The latter ones have exhibited efficient catalytic activity in the asymmetric Mannich reaction. In fact, the use of simple *trans*-(1*R,*2*R*)-aminoindanol (**1c**) as an efficient organocatalyst in the enantioselective synthesis of natural products as the TMC-954 core [12–13], has been recently reported. These examples show the high catalytic potential that this versatile motif exhibits [14].

The concept of bifunctionality has been extensively explored in organocatalysis in the last decade [15–16]. The bifunctional organocatalyst contains two chemical groups that interact simultaneously with the substrates. This mode of activation increases the efficiency of the process, since the interactions favor a selective approach of the reactants. In the transition state, the chiral and rigid aminoindanol scaffold can be involved in different interactions with the substrates due to its capacity to interact through the hydroxy and amino groups. Although the aminoindanol scaffold appears in the structure of different catalysts (providing a suitable way to induce chirality), it is not always directly involved in the bifunctional activation of the substrates [17]. Herein, we show only those cases where the aminoindanol moiety confers bifunctionality to the organocatalysts, interacting with the reactants through both the hydroxy and amino groups.

## Review

### Bifunctional hydrogen-bonding-based organocatalysts

Most of the examples of bifunctional aminoindanol-containing organocatalysts present in literature correspond to catalysts acting through hydrogen bonding, such as thiourea, urea, squaramide, and thioamide frameworks. These have been efficiently employed in a few organocatalytic processes such as Friedel–Crafts alkylations, Michael additions, Diels–Alder reactions and aza-Henry reactions, as discussed below.

#### Friedel–Crafts-type alkylation reaction of indoles

**Scheme 1:**
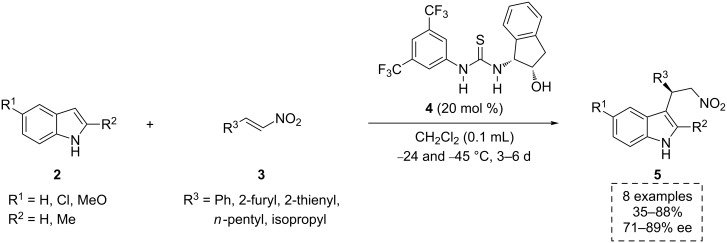
Asymmetric F–C alkylation catalyzed by thiourea **4**.

To the best of our knowledge, the first example of an aminoindanol-containing bifunctional organocatalyst was reported by Ricci and co-workers in 2005 [18]. In this pioneering study, the authors used the easily prepared *cis*-(1*R,*2*S*)-aminoindanol-based thiourea derivative **4** to develop the first organocatalytic enantioselective Friedel–Crafts (F–C) alkylation of indoles, employing nitroalkenes as versatile electrophiles. In the presence of catalyst **4**, the differently functionalized indole derivatives **2** reacted with aryl and alkyl nitroalkenes **3** in dichloromethane at low temperature. This afforded the optically active 2-indolyl-1-nitro compounds **5** (up to 88% yield and up to 89% ee, Scheme 1). These products were found to be valuable synthetic precursors of biologically active compounds such as tryptamines [19–20] and 1,2,3,4-tetrahydro-β-carbolines [21].

**Figure 2:**
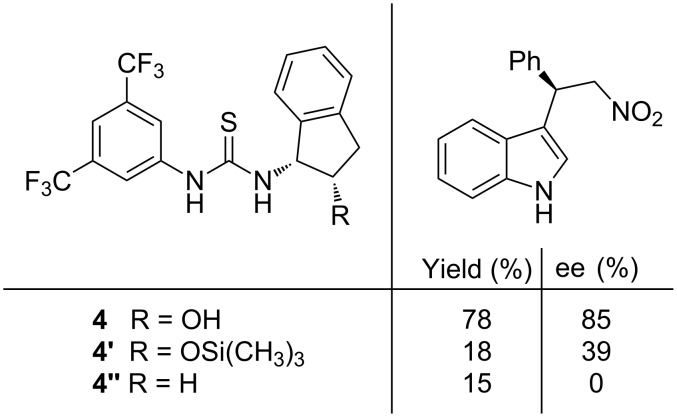
Results for the F–C reaction carried out with catalyst **4** and the structurally modified analogues, **4'** and **4''**.

In order to explain the sense of the asymmetric induction observed in the reaction, some experiments with structurally modified catalysts (**4'** and **4''**) were carried out. The results obtained using indole (**2a**) and β-nitrostyrene (**3a**) supported the importance of the hydroxy group, since low yield and selectivity were observed when this group was trimethylsylilated (**4'**) or was not present (**4''**) in the catalytic structure (Figure 2). Moreover, poor selectivity was also observed using *N*-methylindole, which supported a plausible catalyst–substrate interaction through the indolic proton.

**Figure 3:**
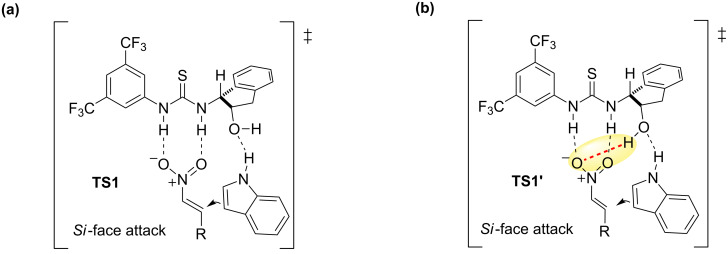
(a) Transition state **TS1** originally proposed for the F–C reaction catalyzed by thiourea **4** [18]. (b) Transition state **TS1’** proposed later, based on computational calculations [22].

The authors proposed then a dual role of catalyst **4** in the activation of the substrates. Thus, in the transition state **TS1** (Figure 3a), the substrates and catalyst would form a ternary complex where the thiourea moiety would activate the nitro group of the nitroalkene through hydrogen bonds. Simultaneously, the oxygen atom of the hydroxy group would interact with the NH of the indole by a weak hydrogen bond, driving the attack to the *Si* face of the nitroalkene in a stereocontrolled manner.

In a recent study of this F–C alkylation, Herrera’s group has provided computational evidence of the reaction pathway, which confirms the proposed bifunctional activation mode played by the thiourea catalyst **4** [22]. Remarkably, an interesting hydrogen-bonding interaction between the hydrogen atom of the hydroxy group and the nitro group was detected in this work (Figure 3b). This could explain the low reactivity (18% yield) and selectivity (39% ee) that the silyl ether-protected catalyst **4''** exhibited (Figure 2).

**Scheme 2:**
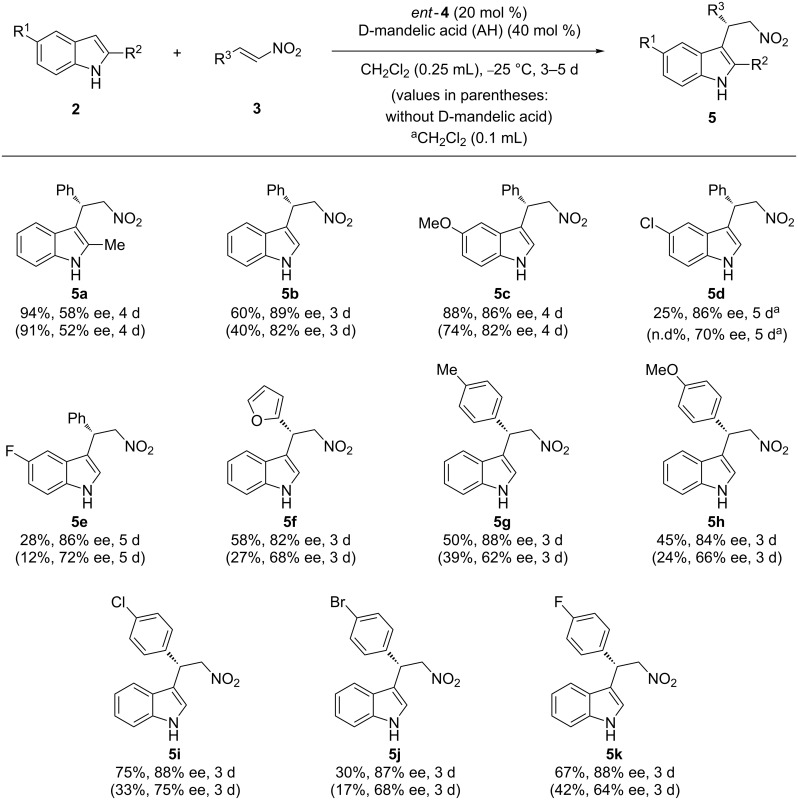
Asymmetric F–C alkylation catalyzed by thiourea *ent*-**4** in the presence of D-mandelic acid as a Brønsted acid additive.

Encouraged by the development of more efficient organocatalytic systems, the same research group explored the influence of external acidic additives in this reaction. The authors envisioned that a cooperative effect between the chiral thiourea organocatalyst and a Brønsted acid (AH) could provide better results in terms of reactivity and enantioselectivity. Thus, in 2011, they published an article where it was proved that the synergic system between the thiourea *ent*-**4** and mandelic acid led to the final products **5** with a significant increase of conversion and enantiomeric excess (Scheme 2) [23].

**Figure 4:**
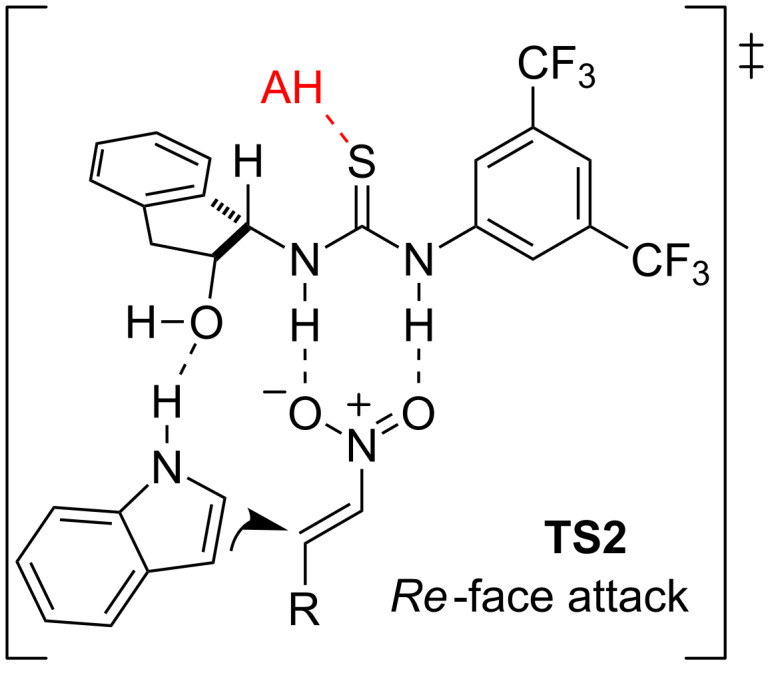
Transition state **TS2** proposed for the activation of the thiourea-based catalyst *ent*-**4** by an external Brønsted acid.

Experimental proofs exploring different catalysts and acids suggested that it is the thiourea which provides the sense of the enantioinduction. Therefore, the authors assumed the bifunctional transition state **TS2**, similar to the above mentioned **TS1**, where the external acid (AH) would only coordinate to the thiourea moiety enhancing its acidity and thus forming a more active catalytic species (Figure 4).

Since the pioneering aminoindanol-containing organocatalyst **4**, reported in 2005 [18], other research groups have studied the possibility of incorporating this scaffold into diverse organocatalysts.

**Scheme 3:**
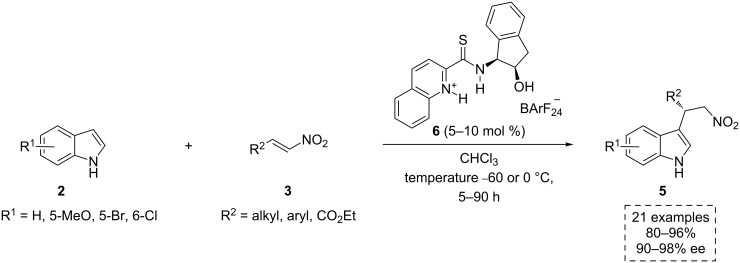
Friedel–Crafts alkylation of indoles catalyzed by the chiral thioamide **6**.

In 2008, Seidel’s group published a new example of an asymmetric addition of indoles to nitroalkenes, employing a novel catalyst design [24]. The authors envisioned that a protonated 2-pyridyl substituent could increase the acidity of the thiourea group through an intramolecular N–H···S hydrogen-bonding interaction (analogous to the C–H···S that exists with the 3,5-bis-trifluoromethylphenyl moiety, commonly used in thiourea-based organocatalysts) [25]. Although this first approach did not provide a significant increase of the enantioselectivity, further modifications of the catalytic structure led to highly active catalysts. Indeed, the best results were obtained with the quinolinium thioamide **6**, where the NH moiety adjacent to the pyridine ring of the analogous thiourea was “removed”. Likely, in this case, the intramolecular hydrogen-bonding interaction described above would yield a negligible stabilization due to the distance between N–H and S moieties. In contrast, it is suspected that both the thioamide N–H as well as the N–H on the quinolinium moiety are engaged in substrate binding, and thus, provide higher yields and selectivity in comparison with the catalyst **4** (up to 96% yield, up to 98% ee) (Scheme 3).

**Scheme 4:**
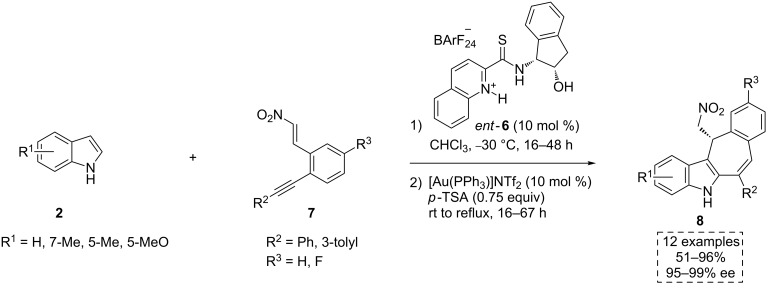
Scalable tandem C2/C3-annulation of indoles, catalyzed by the thioamide *ent*-**6**.

The authors do not comment on whether the catalyst **6** acts in a bifunctional fashion or not, but it is reasonable to assume that the OH group is again involved in the transition state by a possible interaction with the indole derivatives **2**. Indeed, as discussed below, other authors proposed the compound **6** as a plausible bifunctional catalyst. The Enders’ group used its enantiomer (*ent*-**6**) to develop a pioneering scalable one-pot multicatalytic method for the C2/C3-annulation of the indoles **2** (Scheme 4) [26]. In this work, an efficient enantioselective and sequential double Friedel–Crafts alkylation provided direct access to the tetracyclic seven-membered ring containing indoles **8**. These pharmaceutically intriguing compounds exhibit anticancer [27] and antiproliferative activity [28].

**Scheme 5:**
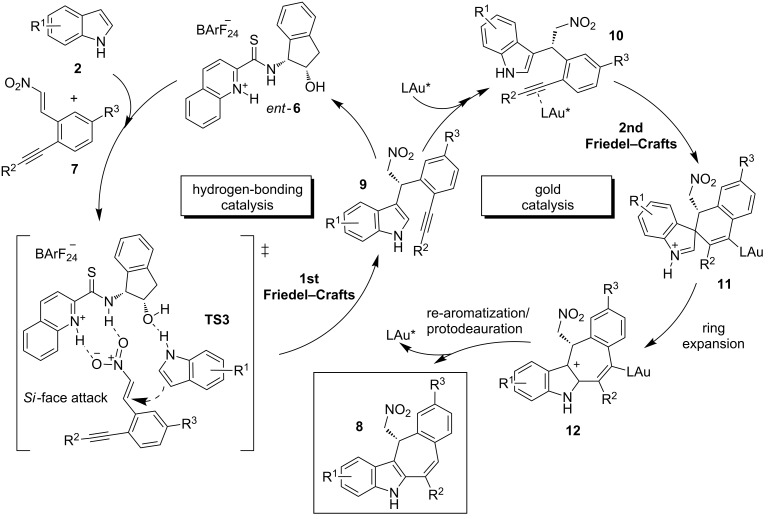
Plausible tandem process mechanism for the sequential, double Friedel–Crafts alkylation, which involves the hydrogen-bonding catalyst *ent*-**6** and gold catalysis and leads to the tetracyclic indoles **8**.

In the first catalytic cycle of the authors’ mechanistic hypothesis, the β-nitroalkene derivatives **7** are proposed to react with the indoles **2** in the presence of the organocatalyst *ent*-**6** to afford the intermediates **9** with excellent enantioselectivity (Scheme 5). Furthermore, a bifunctional activation mode through the transition state **TS3** was proposed. Herein, the NH from the thioamide and the protonated quinoline moiety would activate and fix the nitroalkene framework through hydrogen-bonding interactions. Simultaneously, the oxygen atom of the hydroxy group would orientate the attack of the indole by the *Si* face through the formation of a hydrogen bond with the indolic proton. In the second catalytic cycle, the intermediates **9** would react to give an intramolecular Friedel–Crafts alkylation. The alkyne moiety of **9** would be previously activated by a gold complex in the presence of *p*-toluenesulfonic acid hydrate as the additive. The final tetracyclic indoles **8** are released from the spirocyclic intermediates **11**, following a ring-expansion and rearomatization/final protodeauration cascade process (Scheme 5) [26].

**Scheme 6:**
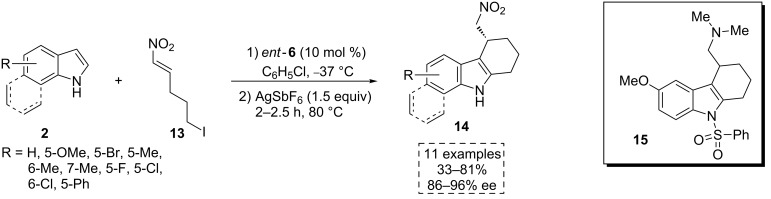
One-pot multisequence process that allows the synthesis of interesting compounds **14**. The pharmacologically active compound **15** can be obtained from the properly substituted product **14**.

**Scheme 7:**
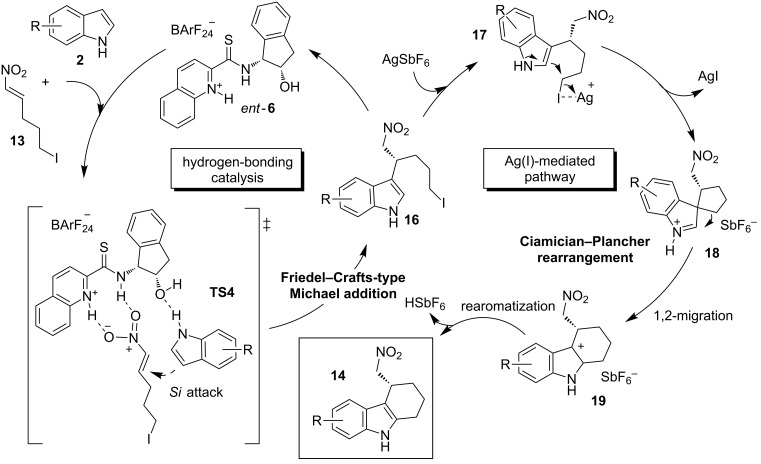
Reaction pathway proposed for the preparation of the compounds **14**.

In 2012, the same group reported an additional example of a one-pot multisequence reaction following a similar mode of activation. This method provided a route to access the enantiomerically enriched tetrahydrocarbazole scaffold-containing compounds **14** (Scheme 6 and Scheme 7) [29]. One of these valuable products is a synthetic precursor of the pharmacologically active compound **15**, used to treat Alzheimer and other central nervous system diseases [30–34].

In the proposed reaction pathway, the nucleophilic addition of the indole derivatives **2** to the nitroalkene **13** progresses in a stereocontrolled manner due to the creation of a ternary complex with the chiral bifunctional thioamide *ent*-**6** (**TS4**, Scheme 7). Herein, the catalyst activates both substrates simultaneously through hydrogen-bonding interactions between the thioamidic NH and the nitro group, and between the hydroxy group and the indolic proton. In the presence of AgSbF_6_, a soft Lewis acid, the stereogenic center-containing intermediates **16** are activated. This triggers an S_N_2-type attack/Ciamician–Plancher rearrangement [35]/rearomatization cascade process, affording the final products **14** (Scheme 7).

**Scheme 8:**
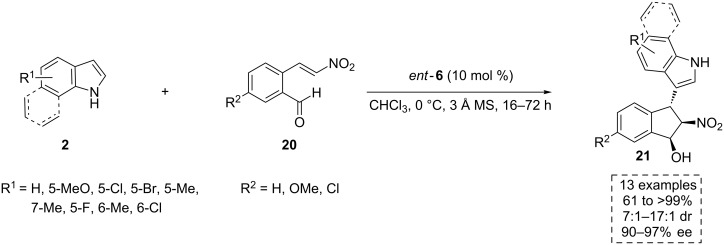
The enantioselective synthesis of *cis*-vicinal-substituted indane scaffolds **21**, catalyzed by *ent*-**6**.

More recently, the same authors also provided an elegant and efficient solution to give direct access to *cis*-vicinal-substituted indane scaffolds through an organocatalyzed asymmetric domino-Michael addition/Henry reaction (Scheme 8) [36]. These heterocyclic products are important chiral building blocks for the synthesis of organocatalytic frameworks and ligands for chiral metal complexes, both with the potential ability to induce chirality. They also belong to a class of privileged pharmaceutical scaffolds and exhibit different biological activities, as it is the case of Crixivan [37–38], an HIV protease inhibitor which has been employed for AIDS treatment. The thermodynamic unfavorable *cis* conformation of these compounds represents a challenge for the development of suitable methods for their synthesis. Interestingly, the chiral thioamide *ent*-**6**, which contains a *cis*-vicinal-substituted indane motif, provided the best choice to this purpose. Therefore, in the presence of such a catalyst, the indole derivatives **2** reacted with 2-(2-nitrovinyl)benzaldehyde derivatives **20** to give the highly functionalized *cis*-1-hydroxy-2-nitroindane-based indole compounds **21** with excellent yield, high selectivity and good diastereomeric ratios (dr) (Scheme 8).

**Scheme 9:**
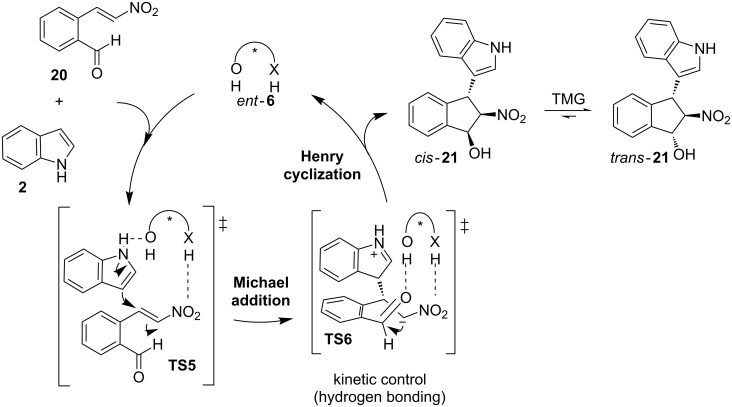
Asymmetric domino procedure (Michael addition/Henry cyclization), catalyzed by the thioamide *ent*-**6** which involves a *cis*-matched transition state (**TS6**) that allows a kinetic control of the second reaction.

In the reaction pathway proposed to explain this domino reaction, both substrates are activated by the catalyst *ent*-**6** through hydrogen-bonding interactions in a bifunctional manner. Thus, the ternary complex formed in the transition state **TS5** leads to an enantioselective Friedel–Crafts-type Michael addition by the attack of indole **2** to the electrophilic prochiral center on the nitroalkene **20** in a stereocontrolled manner (Scheme 9). Afterwards, the hydrogen-bonding interactions are reorganized inside the complex, producing a bifunctional activation of both the nitro and the aldehyde groups through a *cis*-matched transition state **TS6**. It allows a kinetic controlled, enantioselective Henry reaction that leads to the final *cis*-product **21**. The thermodynamically favorable *trans*-product **21** can be obtained through a tetramethylguanidine (TMG)-catalyzed epimerization process.

**Scheme 10:**
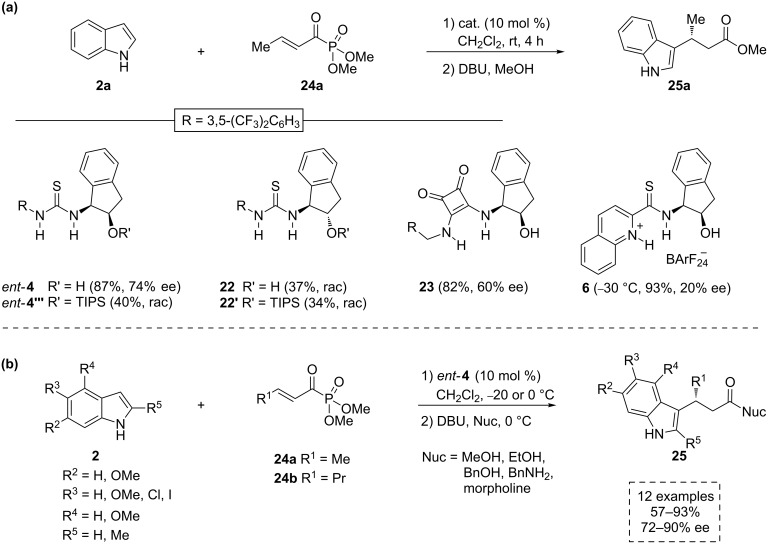
The enantioselective addition of indoles **2** to α,β-unsaturated acyl phosphonates **24**, a) screening of different catalysts and b) optimized conditions using catalyst *ent*-**4**.

Other possible electrophiles have been contemplated in the Friedel–Crafts alkylation of indoles. For instance, Jørgensen’s group studied the use of α,β-unsaturated acyl phosphonates as suitable electrophiles for this kind of reaction, using several bifunctional aminoindanol-based organocatalytic scaffolds as catalysts (Scheme 10) [39]. Hence, the authors demonstrated that acyl phosphonates can be used as efficient hydrogen-bonding acceptors in their activation through hydrogen-bonding catalysis. The corresponding final esters or amides are obtained after proper treatment of the reaction mixture. During the screening of catalysts, the best enantioselectivity (74% ee) was obtained using *ent*-**4** in the addition of indole (**2a**) to the acyl phosphonate **24a**, in dichloromethane at room temperature, with subsequent addition of 1,8-diazabicyclo[5.4.0]undec-7-ene (DBU) and methanol to give the corresponding ester derivative **25a** (Scheme 10a) [39]. The use of the analog squaramide **23** afforded the product with slightly lower selectivity (60% ee) and the Seidel’s thioamide **6** provided better activation (93% yield at −30 °C) of substrates but with an important loss of selectivity (20% ee). The removal of a hydrogen atom from the hydroxy group of the aminoindanol structure (such as in TIPS-ether catalysts *ent*-**4'''** and **22'**) and the loss of *cis* relationship between the hydroxy and amino groups (such as in catalyst **22**) led to racemic mixtures (Scheme 10a). Under optimal conditions, the acyl phosphonates **24** reacted with indoles **2** in the presence of catalyst *ent*-**4** providing the corresponding products **25** with high selectivity (up to 90% ee) (Scheme 10b) [39].

**Figure 5:**
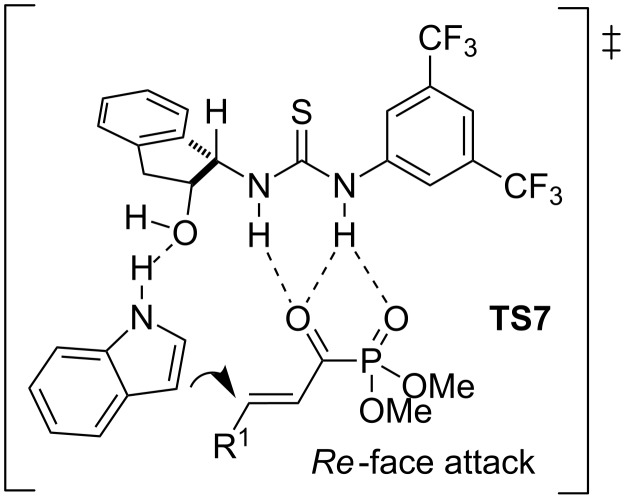
Proposed transition state **TS7** for the Friedel**–**Crafts reaction of indole and α,β-unsaturated acyl phosphonates catalyzed by *ent*-**4**.

Based on the experimental results, the authors proposed a bifunctional mode of activation (**TS7**), where the electrophile is fixed and activated by the thiourea framework through several hydrogen bonds. At the same time, the indole is oriented to attack the *Re* face of the Michael-type acceptor, by weak hydrogen-bonding interaction between the oxygen atom of the hydroxy group and the indolic proton (Figure 5).

**Scheme 11:**
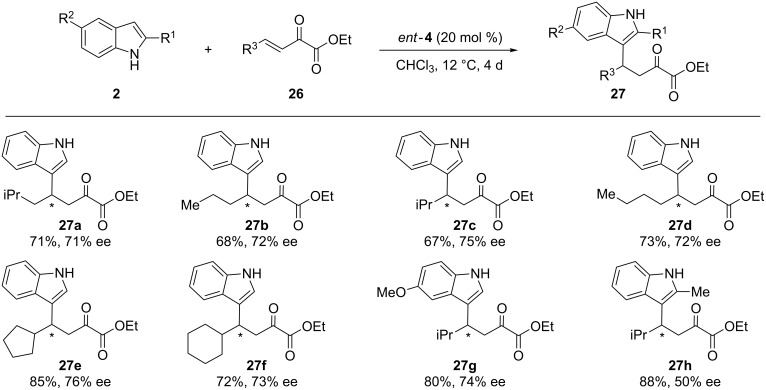
Study of aliphatic β,γ-unsaturated α-ketoesters **26** as substrates in the F–C alkylation of indoles catalyzed by *ent*-**4**.

Recently, the conjugated addition of indole derivatives to β,γ-unsaturated α-ketoesters was explored [40]. To this end, the catalytic activity of several chiral thioureas was studied, revealing the aminoindanol-based thiourea *ent*-**4** as the most suitable catalyst for this process. The authors studied aliphatic derivatives because for this reaction these compounds had been much less explored than the aromatic ones. Thus, the different aliphatic β,γ-unsaturated α-ketoesters **26a**–**f** reacted with the substituted indoles **2** in the presence of *ent*-**4** to achieve the corresponding adducts **27** with good yields and enantioselectivities (up to 88% yield, up to 76% ee) (Scheme 11).

**Figure 6:**
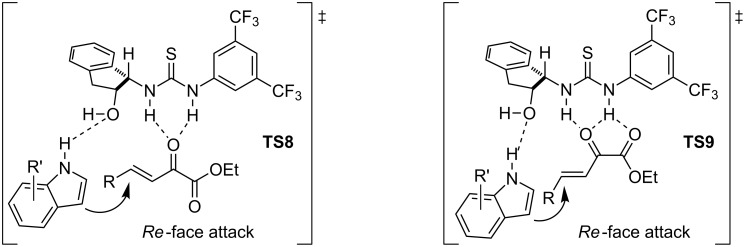
Possible transition states **TS8** and **TS9** in the asymmetric addition of indoles **2** to the β,γ-unsaturated α-ketoesters **26** catalyzed by *ent*-**4**.

Although the absolute configuration was unknown at that point, the authors envisioned a plausible reaction pathway based on previously reported transition states (Figure 6). The catalyst *ent*-**4** would activate and fix the electrophile through several hydrogen-bonding interactions with the NH groups of the thiourea. Simultaneously, the hydroxy group would be involved in the activation of the nucleophile, establishing a hydrogen bond with the indolic proton. This would conduct its attack over the *Re* face of the β,γ-unsaturated α-ketoesters, producing the addition in a stereocontrolled fashion. Some additional experimental proofs provided in the article supported this hypothesis [40].

#### Michael addition to α,β-unsaturated compounds

Fernández, Lassaleta and co-workers provided an elegant, versatile and mild umpolung strategy, which leads to key synthetic precursors using the thiourea *ent*-**4**. In this study, an organocatalytic enantioselective addition of nucleophilic *N*,*N*-dialkylhydrazones to electron-deficient β,γ-unsaturated α-ketoesters was reported (Table 1) [41]. In the presence of catalyst *ent*-**4**, 1-methyleneaminopyrrolidine (**28**) reacted with the different β,γ-unsaturated α-ketoesters **26** in dichloromethane at low temperature to give the corresponding products **29**, which are useful masked 1,4-dicarbonyl compounds with moderate to high yield and high selectivity, after moderate reaction times (Table 1).

**Table 1:** Asymmetric addition of 1-methyleneaminopyrrolidine (**28**) to β,γ-unsaturated α-ketoesters **26**, catalyzed by *ent*-**4**.

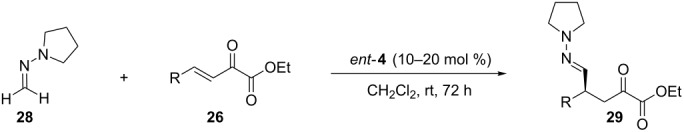				

Entry	**26** (R)	Temp. (ºC)	Yield **29** (%)	ee **29** (%)

1	Me	−60	60	80

2	iPr	−45	80	78

3	iBu	−45	75	78

4	*n*-C_5_H_11_	−60	61	70

5	(CH_3_)_3_CH_2_	−45	64	58

6	Cy	−45	82	72

**Figure 7:**
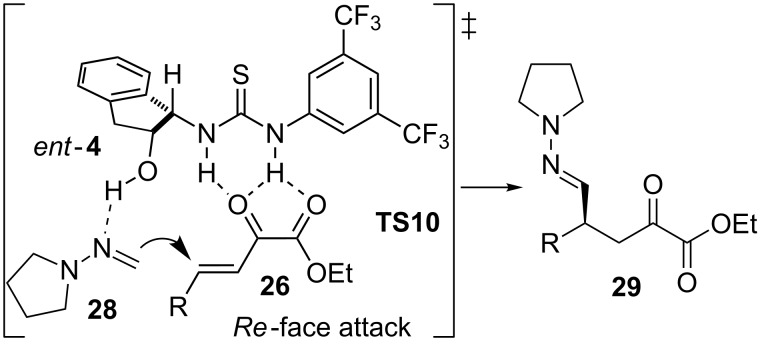
Transition state **TS10** proposed for the asymmetric addition of dialkylhydrazone **28** to the β,γ-unsaturated α-ketoesters **26** catalyzed by *ent*-**4**.

The authors proposed the plausible transition state **TS10**, where the acidic hydrogen atoms of the thiourea could activate the β,γ-unsaturated α-ketoesters **26**. Simultaneously, the hydrogen atom of the hydroxy group would coordinate and direct the hydrazone **28** to the *Re* face of the esters **26** in order to afford the absolute configuration found in the final products **29** of this process (Figure 7).

Another example of the bifunctional action of the indanol-based thiourea **4** was reported by Sibi’s group. There, 100 mol % of this compound was employed in the enantioselective conjugate addition of the hydroxylamine derivatives **31** to the enoates **30**, affording the final products **32** with good yield (up to 98%) and high enantiomeric excess (up to 98% ee). This provided an efficient method that allows the preparation of biologically interesting β-amino acid derivatives (Table 2) [42].

**Table 2:** The enantioselective addition of the hydroxylamine derivatives **31** to the enoates **30** promoted by **4**.

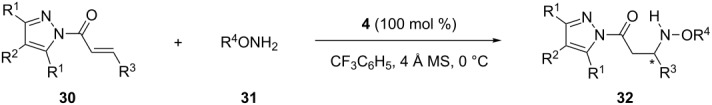					

Entry	**30** (R^1^, R^2^, R^3^)	**31** (R^4^)	Time (h)	Yield **32** (%)	ee **32** (%)

1^a^	Me, H, Me	PhCH_2_	24	75 (**32a**)	71

2^a,b^	Me, H, Me	PhCH_2_	168	63 (**32a**)	71

3	Me, H, Me	PhCH_2_	72	82 (**32a**)	87

4^a^	Me, Br, Me	PhCH_2_	24	85 (**32b**)	61

5^a^	Ph, H, Me	PhCH_2_	14	76 (**32c**)	45

6^a^	Ph, Br, Me	PhCH_2_	12	72 (**32d**)	31

7	Me, H, Me	Ph_2_CH	96	86 (**32e**)	89

8	Me, H, CO_2_Et	Ph_2_CH	96	50 (**32f**)	94

9	Me, H, CO_2_Et	TBDMS	96	42 (**32g**)	90

10	Me, H, Et	Ph_2_CH	168	92 (**32h**)	91

11	Me, H, *n*-Pr	Ph_2_CH	138	84 (**32i**)	88

12	Me, H, iPr	Ph_2_CH	216	68 (**32j**)	90

13	Me, H, *c*-C_6_H_11_	Ph_2_CH	288	59 (**32k**)	89

14^b^	Me, H, CH_2_OPMP	Ph_2_CH	24	98 (**32l**)	98

15^a^	Me, H, Ph	PhCH_2_	72	19 (**32m**)	67

16	Me, H, Me	TBDMS	120	82 (**32n**)	94

^a^Reaction carried out at room temperature. ^b^30 mol % of catalyst **4**.

**Scheme 12:**
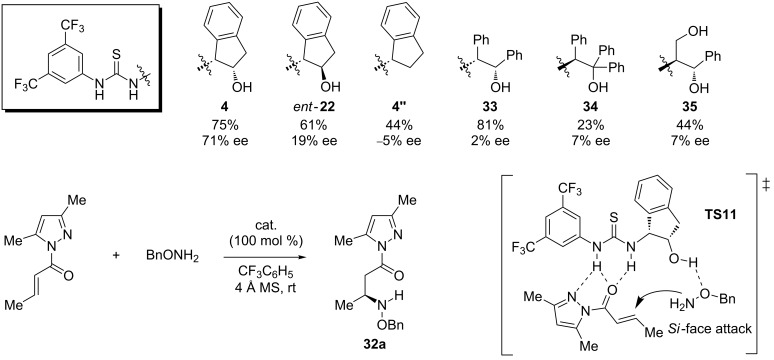
Different β-hydroxylamino-based catalysts tested in a Michael addition, and the transition state **TS11** proposed for this reaction catalyzed by **4**.

In this work, the authors compared the results achieved by means of **4** with other urea- and thiourea-based organocatalysts in order to understand the effect of the acidity, the structural rigidity, and the bifunctionality of the promoter. These reactions were performed in trifluorotoluene at room temperature with the Michael acceptor **30** (R^1^, R^2^, R^3^ = Me, H, Me) and *O*-benzylhydroxylamine (**31**, R^4^ = PhCH_2_), using a stoichiometric amount of the chiral activator and MS 4 Å as an additive. Some of the reported experiments supported the ability of the *cis*-2-aminoindanol structure to provide an adequate scaffold to induce chirality. In contrast, the catalysts *ent-***22** (with the *trans*-2-aminoindanol) or **4''** (with the aminoindane motif) and the flexible analogues **33**–**35**, provided lower enantioselectivities or led to nearly racemic mixtures (Scheme 12). In the proposed transition state **TS11**, the α,β-unsaturated substrate is activated by an acidic thiourea template. Moreover, the hydroxylamine derivative is simultaneously oriented to attack the *Si* face of the Michel acceptor, through its interaction with the hydroxy group of the aminoindanol framework. In this case, a pyrazole moiety presents additional H-bond acceptor sites. These could play an important role in fixing the substrate to the catalyst and favoring a more rigid transition state (**TS11**) and thus leading to better selectivity (Scheme 12). The absolute configuration is only given for the compounds **32a**–**d**, being *S*.

**Scheme 13:**
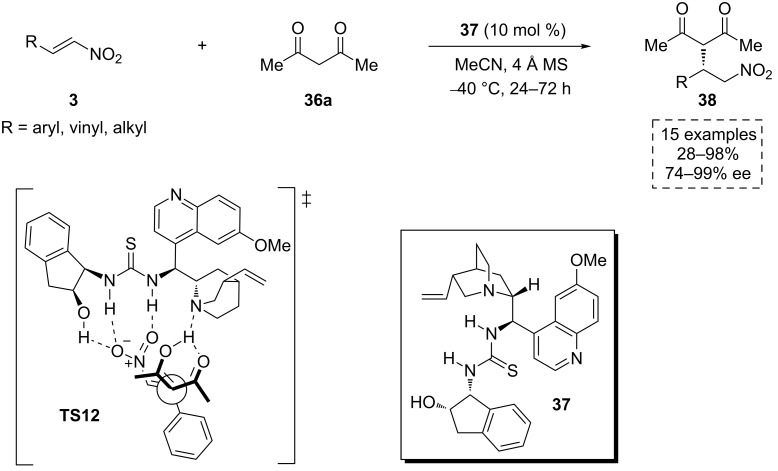
Enantioselective addition of acetylacetone (**36a**) to nitroalkenes **3**, catalyzed by **37** and the proposed transition state **TS12**.

Later, He and co-workers reported the use of several chiral multiple hydrogen-bond donating tertiary amine-based organocatalysts in the asymmetric addition of acetylacetone (**36a**) to the β-nitroalkenes **3**. They found the thiourea **37** as a highly suitable catalytic structure to induce chirality in this process (Scheme 13) [43]. Under optimal conditions, this method provided highly enantioenriched γ-nitrocarbonyl compounds **38**, which are versatile synthetic intermediates for the preparation of diverse chiral scaffolds.

Once again, a bifunctional activation mode as the origin of the asymmetric induction was proposed. In the plausible transition state **TS12**, acidic hydrogen atoms from both hydroxy and thiourea moieties would activate and fix the nitroalkene. Simultaneously, the tertiary amine of the cinchona framework would deprotonate the acidic proton of acetylacetone (**36a**), driving the attack of the nucleophile. The chiral environment present in the resulting ternary complex would confer the proper facial selectivity to afford the observed absolute configuration in the final products **38**.

**Scheme 14:**
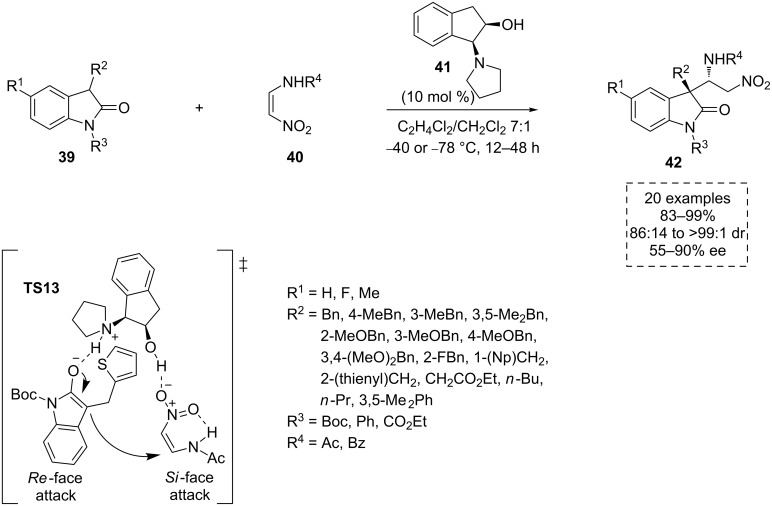
Addition of 3-oxindoles **39** to 2-amino-1-nitroethenes **40**, catalyzed by **41**.

At the same time, Yuan and co-workers developed an interesting example of a scalable asymmetric Michael addition of 3-substituted oxindoles **39** to the protected 2-amino-1-nitroethenes **40**, using the bifunctional tertiary amine aminoindanol-based organocatalyst **41** (Scheme 14) [44]. This catalytic study provides a straightforward synthetic route of the highly functionalized α,β-diamino-3,3’-disubstituted-oxindoles **42**. These are key intermediates for the preparation of biologically and pharmacologically attractive compounds, such as (+)-alantrypinone [45], (−)-serantrypinone [46] and (−)-lapatin [47]. In the presence of the catalyst **41** (10 mol %), a broad scope of the oxindoles **39** reacted to give the quaternary stereocenters-containing products **42** with high diastereoselectivity (up to > 99:1 dr) and enantioselectivity (up to 90% ee).

A bifunctional role played by the catalyst was again envisioned by the authors. In the transition state **TS13** the tertiary amine group of the catalyst would activate the resulting enolized oxindole reagent **39** via deprotonation. Thus, **39** would be disposed to attack by its *Re* face to the *Si* face of the nitroethene derivative **40**. Simultaneously, the latter would be fixed and activated by a hydrogen-bonding interaction with the hydroxy moiety of the catalyst, in its *Z* form, which is stabilized due to an intramolecular hydrogen bond (Scheme 14).

**Scheme 15:**
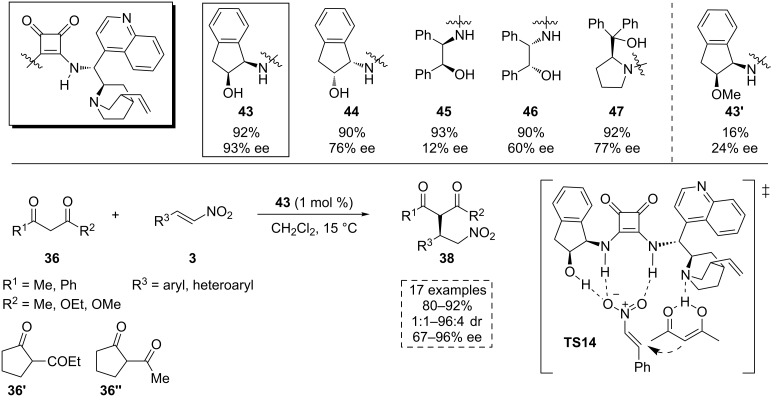
Michael addition of 1,3-dicarbonyl compounds **36** to the nitroalkenes **3** catalyzed by the squaramide **43**.

In 2012, Dong and co-workers studied the catalytic activity of several β-amino alcohol-based squaramide organocatalysts involved in the Michael addition of acetylacetone (**36a**) to β-nitrostyrene (**3a**) in dichloromethane at 15 °C (Scheme 15) [48]. Although high yields were obtained in all cases, the best enantioselectivity was provided by the bifunctional *cis-*aminoindanol-based squaramide **43**. Under these conditions, several 1,3-dicarbonyl compounds **36** reacted with many different nitrostyrene derivatives **3** with very low catalytic charge (1 mol %), affording a broad scope of the enantiomerically enriched β-nitroalkyl products **38**. A possible drawback of the method would be the low diastereoselectivity generally achieved for the nonsymmetrical 1,3-dicarbonyl compounds **36**.

In order to understand the role of the catalyst, the hydroxy group of the squaramide **43** was methylated (**43'**). Its catalytic activity was tested in the reaction of acetylacetone (**36a**) and β-nitrostyrene (**3a**), leading to very low enantiomeric excess (24% ee). This fact suggested the important role played by the hydroxy group in the activation and in the chiral induction of the process. The authors proposed the transition state **TS14**, where the NH groups and the OH group of the squaramide would coordinate to the nitroalkene **3** through hydrogen-bonding interactions with the nitro group. Simultaneously, the amine in the cinchona alkaloid would activate the 1,3-dicarbonyl compound **36** (Scheme 15). We would like to remark that although the authors indicated that the *S* enantiomer is obtained in their final products, they depicted the *R* configuration, as is drawn in the Scheme 15.

#### Aza-Henry reaction

**Scheme 16:**
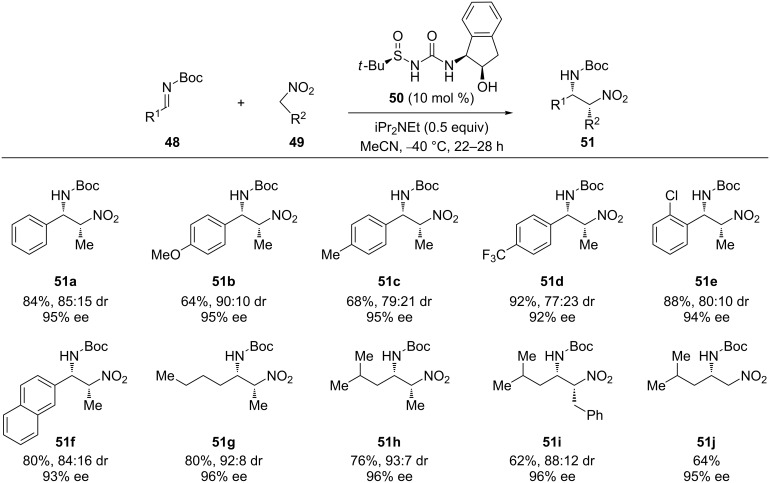
Asymmetric aza-Henry reaction catalyzed by the aminoindanol-derived sulfinyl urea **50**.

Ellman’s group designed a set of pioneering (thio)urea scaffold-containing hydrogen-bonding organocatalysts with an *N*-sulfinyl moiety. As previously demonstrated, this chemical group increased the acidity of the catalyst and also served as a chiral controller [49–54]. Hence, in the presence of the catalyst **50** and diisopropylethylamine, a wide scope of the *N*-Boc-protected imines **48**, including aliphatic ones, reacted with an excess of the nitroalkanes **49** at low temperature. This afforded the corresponding products **51** with high yield, diastereomeric ratio and excellent enantioselectivity (Scheme 16) [55].

**Figure 8:**
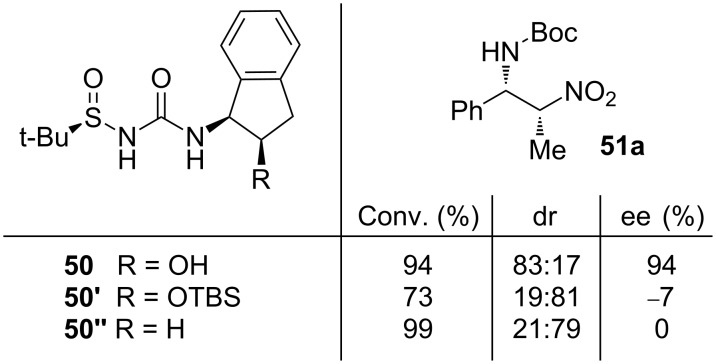
Results for the aza-Henry reaction carried out with the structurally modified catalysts **50**–**50''**.

Some experimental results using the differently substituted aminoindane-derived sulfinyl ureas **50–50''** showed the important effect of the indanol framework in the diastereo- and enantio-selectivity of the process. The catalysts **50'** (with the TBS-protected hydroxy group (TBS, *tert*-butyldimethylsilyl)) and **50''** (without the hydroxy group) exhibited poor enantioselectivity. These effects may suggest and support the bifunctional role played by the catalyst (Figure 8).

#### Diels–Alder reaction

**Scheme 17:**
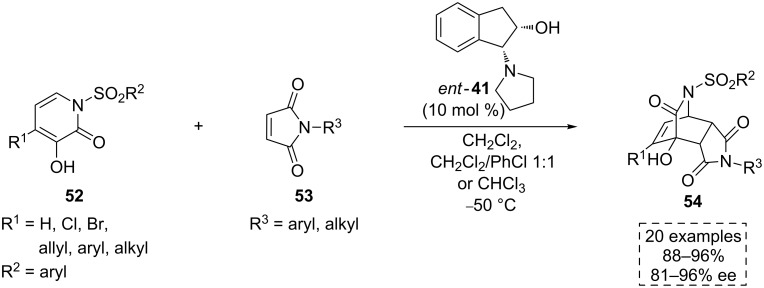
Diels–Alder reaction catalyzed by the aminoindanol derivative *ent*-**41**.

An important contribution in the construction of highly substituted carbocyclic compounds was disclosed by Tan’s group in 2009. In this work, the asymmetric Diels–Alder (D–A) reaction between the *N*-sulfonamide-3-hydroxy-2-pyridone-based dienes **52** and different dienophile substrates was developed using the bifunctional *cis*-2-trialkylaminoindanol organocatalyst *ent*-**41** [56]. We show herein the reactivity of this family of dienes with several substituted maleimides **53**, which in the presence of the above mentioned catalyst, afforded the highly substituted *endo*-adducts **54** with high yield and enantiomeric excess (Scheme 17). In this approach, the *cis* orientation of the hydroxy and cyclopentylamine groups of the catalyst was crucial to achieve high enantioselectivity.

### Aminocatalysis

**Scheme 18:**
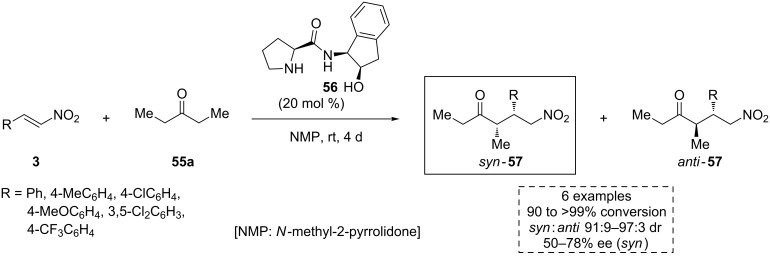
Asymmetric Michael addition of 3-pentanone (**55a**) to the nitroalkenes **3** through aminocatalysis.

Although aminoindanol-derived catalysts have been scarcely used in aminocatalysis, some relevant examples have been found in the literature, especially in the enantioselective addition of ketones to nitroalkene compounds. In this context, Alonso, Nájera and co-workers designed different alcohol-amino-derived prolinamide organocatalysts and in 2006 published an organocatalyzed direct asymmetric Michael addition of 3-pentanone (**55a**) to the nitrostyrenes **3** [57]. The corresponding *syn*-adducts **57** were obtained with excellent conversion, diastereomeric ratio and high enantiomeric excess when the *cis*-aminoindanol-based prolinamide **56**, acting as bifunctional recyclable catalyst, was used (Scheme 18).

**Scheme 19:**
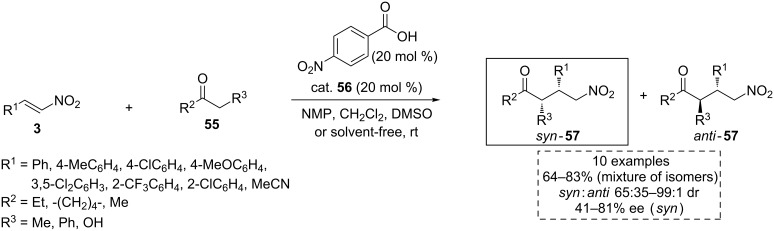
Substrate scope extension for the asymmetric Michael addition between the ketones **55** and the nitroalkenes **3** through aminocatalysis.

Later, based on this previous work, the same research group extended the methodology to different ketones **55**, rendering the *syn*-products **57** with excellent yield and high selectivity (Scheme 19) [58].

**Scheme 20:**
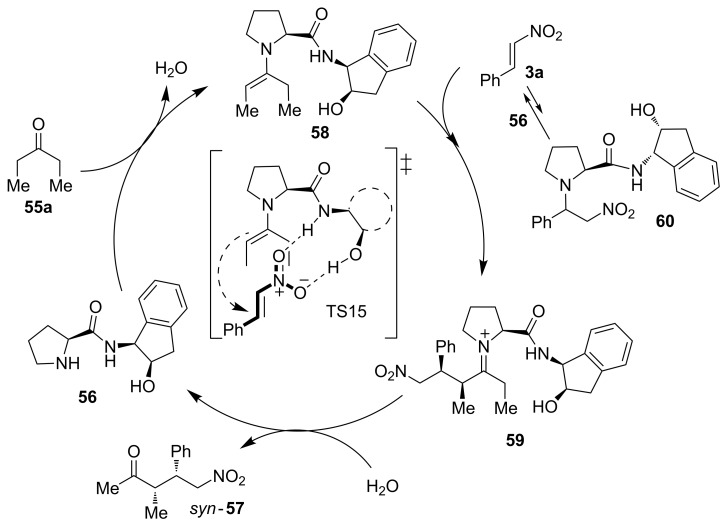
A possible reaction pathway in the presence of the catalyst **56** and the plausible transition state **TS15** proposed for this reaction.

In this case, the hydroxy group seems again to play an important role in the activation of the substrates, as well as in the selectivity of the process. The rigidity of the hydroxylamino moiety represents another important factor, where aminoindanol was the most appropriate scaffold for this asymmetric methodology among the catalysts tested. Based on the experimental results and computational calculations (DFT and B3LYP76-31G*), the authors proposed a reaction mechanism in which the catalyst **56** acts in a bifunctional way following the route depicted in Scheme 20. Thus, Michael addition of the enamine **58**, formed from 3-pentanone (**55a**) and the catalyst **56**, to the nitroalkene **3a** takes place leading to the intermediate **59**. The last step of the catalytic cycle involves the regeneration of the catalyst by hydrolysis, enabled by the small amount of water present in the solvent.

The transition state **TS15** based upon Seebach’s model [59] was envisioned as a plausible activation mode to explain the high asymmetric induction observed and the *syn*-diastereoselectivity exhibited by the catalyst **56**. First, the activation of the ketone via enamine formation is produced. Furthermore, the acidic hydrogen atoms of the amide and the hydroxy groups present in the catalyst would activate and orientate the nitroalkene by hydrogen-bond formation. Thus, the attack of the formed enamine to the *Re* face of the nitroalkene is favored (Scheme 20). In this way, this example shows an efficient combination of covalent and non-covalent interactions in an interesting bifunctional activation mode.

## Conclusion

The design, synthesis and application of catalysts acting in a bifunctional manner is a hot topic in the field of organocatalysis and thus widely investigated. Generally, this particular mode of activation allows the enhancement of both the reactivity and the selectivity of the processes, due to the generation of a more rigid transition state. Among the different ways of conferring this bifunctional character to the catalysts, the incorporation of the aminoindanol core into their structure has shown to be a very suitable method. In most of the examples gathered herein, this can be explained due to the presence of a hydroxy group in the catalyst that normally is able to interact with at least one of the substrates of the reaction, hence facilitating the approach of the reactants in a selective fashion. In many cases, this bifuntional role of the catalyst has been supported with experimental results and sometimes with computational calculations. This smart strategy has allowed the preparation of highly efficient organocatalysts, ranging from very simple structures to more complex ones. These are mainly hydrogen-bonding catalysts, but there is also an example of an aminoindanol-containing aminocatalyst. A broad variety of reactivities has been successfully covered, such as Friedel–Crafts alkylation, Michael addition, Diels–Alder and aza-Henry reactions. However, further exploration into the development of new bifunctional organocatalysts using aminoindanol or another appropriate scaffold and their application in different chemical processes still needs to be performed.
